# Incidence of and survival after surgery for metastatic spine disease: a nationwide register-based study between 1997 and 2020 from Finland

**DOI:** 10.2340/17453674.2025.43264

**Published:** 2025-03-10

**Authors:** Leevi A TOIVONEN, Ville PONKILAINEN, Jussi P REPO, Ville M MATTILA

**Affiliations:** Department of Orthopedics and Traumatology, Unit of Musculoskeletal Diseases, Tampere University Hospital and Tampere University, Tampere, Finland

## Abstract

**Background and purpose:**

Information on metastatic spine disease (MSD) based on nationwide data on trends and postoperative survival is limited but is needed to optimize treatment in this population. We aimed to assess the incidence of and survival rates after MSD surgery.

**Methods:**

This retrospective nationwide register-based study combined data from the Finnish Cancer Registry, Finnish Care Register for Health Care, and the Finnish Cause of Death Register from 1997 to 2020. Surgeries were identified using diagnosis and procedural codes, with primary spine pathologies excluded. Incidence rates were calculated per 100,000 inhabitants and adjusted for age and sex. Survival analysis was conducted using the Kaplan–Meier estimator.

**Results:**

1,845 patients underwent 1,992 surgeries, with a mean age of 65 years; 58% were men. The most common primary cancers were prostate cancer (15.1%), breast cancer (11.6%), and myeloma (10.6%). The incidence of MSD surgery increased by 87%, from 1.05 to 1.97 per 100,000 person-years. Surgery increased most among patients aged 70–79 years. Over the same period, the 6-month survival remained fairly stable. The overall survival probabilities were 57% (95% confidence interval [CI] 54–59) at 1 year, 44% (CI 42–46) at 2 years, 28% (CI 26–30) at 5 years, and 18% (CI 16–20) at 10 years. The 1-year survival was highest in patients with breast cancer at 75% (CI 69–81) and lowest in patients with kidney cancer at 45% (CI 38–53) and prostate cancer at 47% (CI 42–53).

**Conclusion:**

Finnish nationwide data showed an increase in MSD surgery between 1997 and 2020 with a stable postoperative survival of 57% (CI 48–69) to 76% (CI 66–89) at 6 months.

The prevalence of cancer patients is increasing with the incidence of aging populations and improvements in oncologic treatments that translate into higher survival [1,2]. As a result, spinal metastases are increasing and up to 10% of spinal metastases progress to symptomatic spinal cord compression and/or instability, usually eliciting the need for surgery [3].

Surgical management of spinal metastases increased rapidly after Patchell et al. demonstrated the superiority of surgery plus radiotherapy over radiotherapy alone in preserving ambulation [4]. Previously, surgery often consisted of simple decompression of the spinal cord, frequently in emergency settings. Since then, fusion techniques have become the mainstay of spinal metastasis surgery. More recently, less invasive instrumentation and access techniques have gained popularity. These advancements reduce the surgical burden, making procedures more tolerable for older populations [5,6].

Wright et al. reported 1-year survival rates at 53% but slightly increasing long-term survival rates in a prospectively collected series from 22 specialized centers [7]. Using the Swedish National Spine Surgery Register, Swespine, Carrwik et al. reported ae median estimated survival rate of 6.2 months after surgery for spinal metastases in Sweden between 2006 and 2018 [8]. Bhanot et al. reported different directions of survival trends after surgery for spine metastases based on primary pathology in a population-based registry from Ontario [9]. To date, significant variability in surgical strategies is expected between centers managing metastatic spinal disease [10]. It remains unclear whether the advancements reported from specialized centers are applicable nationwide or in broader contexts.

Our primary aim was to report incidence rates and survival after surgery for spinal metastases based on nationwide register data. Our secondary aim was to determine incidence rates and predicted survival for the most common primary cancer diagnoses associated with spine metastasis.

## Methods

### Study design

This retrospective study was based on data derived from 3 nationwide, population-based registers:

1. The Finnish Cancer Registry (FCR) is one of the oldest in the world with excellent coverage and validity for solid tumors [11]. It provided data on age, sex, date of birth, date of cancer diagnosis, principal organ, and diagnosis (ICD 10) codes.
2. The Finnish Care Register for Health Care contains hospital inpatient data with excellent coverage and accuracy [12,13]. Hospitalization-related diagnosis codes, procedural codes, and dates were collected.
3. The Finnish Cause of Death Register, maintained by Statistics Finland, was used for the dates and causes of death. The registry covers all registered deaths of Finnish inhabitants.

The study is reported according to STROBE guidelines.

### Sample formation

We identified spinal metastasis surgeries using the following algorithm: all patients in the FCR by the end of 2021 were screened for spine surgery-related hospitalizations from 1997 to 2020. The spine surgery-related NOMESCO procedural codes used in the screening are listed in Table S1 (see Supplementary data). To be included in the study, potential candidates had to have (a) a cancer-related diagnosis at the hospitalization, or (b) a new cancer registry record within 1 year of surgery. Moreover, diagnosis and procedural codes referring to primary spinal pathologies, disc herniation surgeries, and spondylosis surgeries (Table S2, see Supplementary data) were excluded from the study sample.

### Statistics

Continuous variables were reported as the median with interquartile range (IQR) for skewed distributions and as the mean with standard deviation (SD) for normally distributed variables. The incidence rates were calculated from annual mid-populations, which were obtained from the National Population Register (Official Statistics of Finland). These rates were calculated per 100,000 inhabitants and adjusted for age using the 10-year age and sex distribution of the Finnish population. Kaplan–Meier (KM) survival analysis was used to assess the survival probability with 95% confidence intervals (CI), for patients who had undergone spine metastasis surgery. The follow-up period was defined as follows: observations were censored at the end of the follow-up period (December 30, 2020), while death prior to this date was considered an event. Incidence rates were calculated based on the number of surgeries, and survival analysis was performed using the first metastasis surgery per patient.

Subgroup analyses were conducted to evaluate incidence rates stratified by age, type of surgery, and underlying disease. Given the limited number of cases, the rates were interpreted as 5-year rolling means. We focused on the most prevalent diseases associated with spinal metastasis, including prostate, breast, kidney, lymphoma, myeloma, and lung cancers. All changes in incidence were reported as relative changes (%). As there was no missing data in the dataset, imputation or other methods for handling missing values were unnecessary. Statistical analyses were performed using R version 4.1.1 (R Foundation for Statistical Computing, Vienna, Austria).

### Ethics, data sharing, use of AI, funding, and disclosures

The Finnish Health and Social Data Permit Authority (FinData) granted a study permit (THL/3527/14.02.00/2023) and collected data from the registers. Pseudonymization was carried out by FinData, and the authors did not have access to the pseudonymization key, which was securely held by FinData. Under Finnish law, written informed consent was not required due to the retrospective nature of the register-based study and absence of direct contact with patients. The data supporting the findings of this study are available from Findata with permission, but the authors do not have the authority to share the data. AI tools were not used in this submission. This study received funding from State funding for university-level health research, Tampere University Hospital, Wellbeing Services county of Pirkanmaa. The authors declare no conflicts of interest. Complete disclosure of interest forms according to ICMJE are available on the article page, doi: 10.2340/17453674.2025.43264

## Results

**Figure 1:**
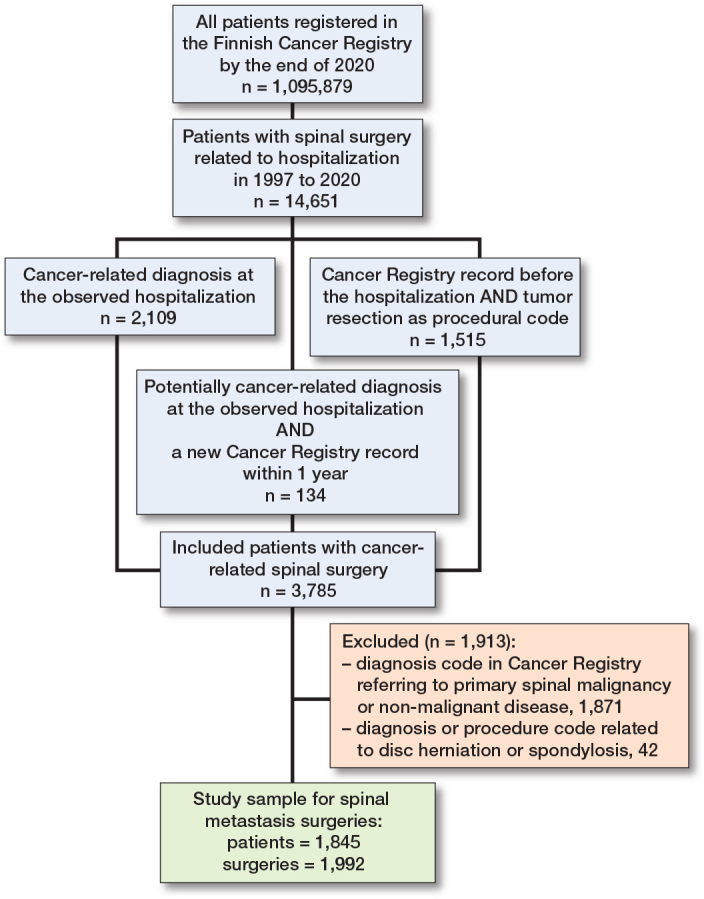
Study flowchart.

The data included 1,845 patients who underwent 1,992 surgeries, with an average of 1.1 operations per patient (Figure 1). The median age at first surgery was 64.6 years (IQR, 56.1–73.6), and 58% of the patients were men. The most common primary cancer diagnoses during the study period were prostate cancer (n = 300, 15.1%), breast cancer (n = 231, 11.6%), and myeloma (n = 211, 10.6%). The distribution of primary cancer diagnoses in the “other” group is shown in Table 1.

**Table 1:** Distribution of primary cancer diagnoses in the “other” group

Primary tumor site	n (%)
Soft tissue/other	182 (20)
Respiratory or thoracic	143 (16)
Gastrointestinal tract	125 (14)
Unspecified	58 (6.5)
Lip, oral cavity, and pharyngeal	56 (6.3
Endocrine	51 (5.7)
Female genital organs	49 (5.5)
Other	36 (4.0)
Bone	33 (3.7)
Unknown	33 (3.7)
Skin	31 (3.5)
Urinary organs	29 (3.3)
Central nervous system	27 (3.0)
Leukemia	22 (2.5)
Eye	6 (0.7)
Male genital organs	6 (0.7)
Immunoproliferative	3 (0.3)

### Incidence rates

**Figure 2:**
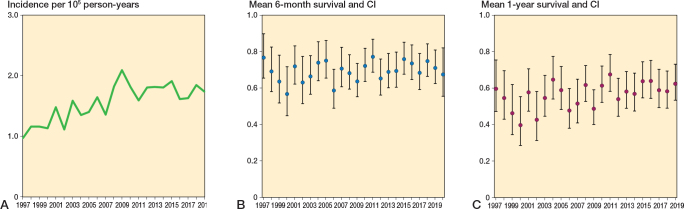
A. Incidence of spine metastasis surgery in Finland from 1997 to 2020. B. Mean 6-month survival with 95% confidence intervals (CI) for patients operated on each year, analyzed using Kaplan–Meier analysis. C. Mean 1-year survival with CI of patients operated on per year, analyzed using Kaplan–Meier analysis.

The age- and sex-adjusted incidence of surgery for spine metastasis increased from 1.05 per 100,000 person-years in 1997 to 1.96 per 100,000 person-years in 2018, an increase of 87% (Figure 2A). During the same period, the 6-month survival rate fluctuated between 57% (CI 48–69) in 2006 and 76% (CI 66–89) in 2004 and 2011 (Figure 2B). The 1-year survival rates ranged from 67% (CI 58–78) in 2011 to 40% (CI 28–55) in 2000 (Figure 2C). However, the general trend in 6-month and 1-year postoperative survival remained fairly stable.

**Figure 3:**
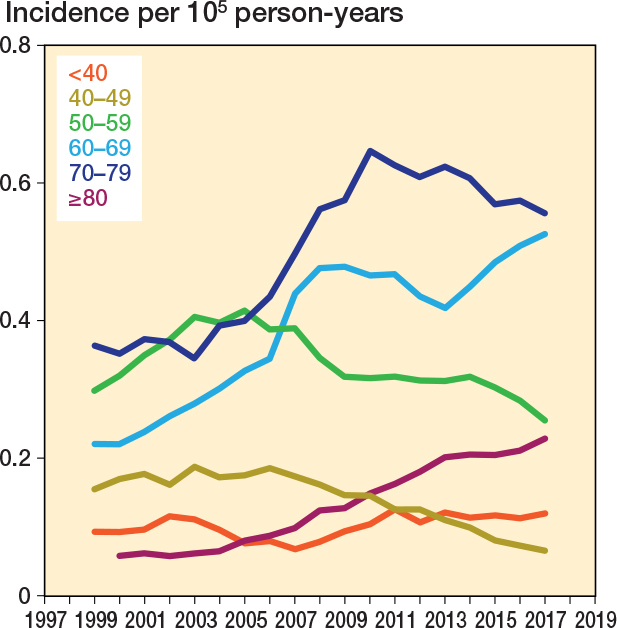
5-year rolling mean incidence of spine metastasis surgery in Finland from 1997 to 2020 stratified by patient age.

The incidence increased most rapidly in patients aged 70–79 years (Figure 3). Between 1999 and 2017, the 5-year rolling mean incidence increased from 0.22 to 0.52 per 100,000 person-years (136% increase) in patients aged 70–79 years.

**Figure 4:**
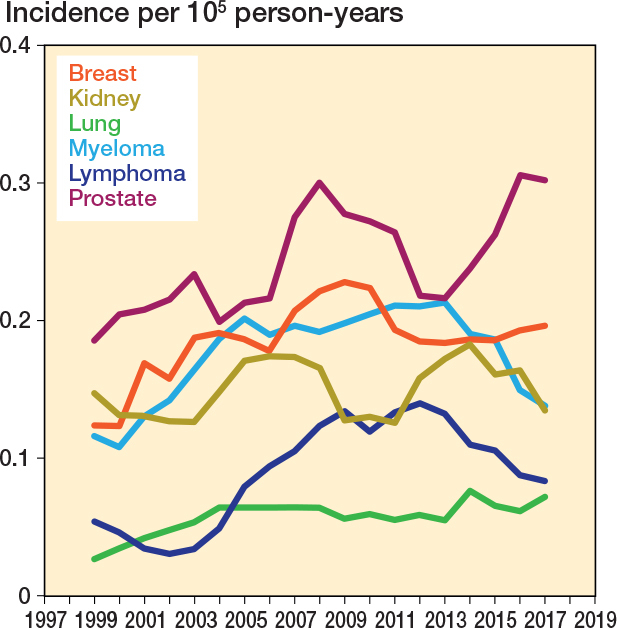
5-year rolling mean incidence of spine metastasis surgery in Finland from 1997 to 2020 stratified by primary disease. Only the 6 most common diseases were included.

**Figure 5:**
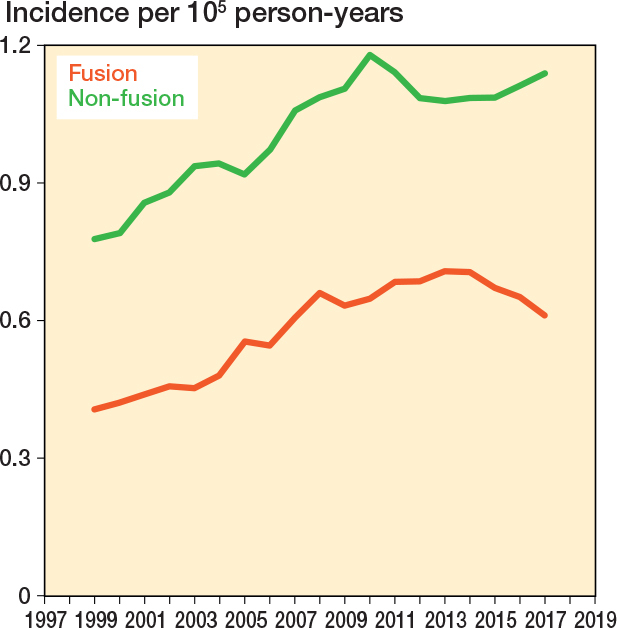
5-year rolling mean incidence of spine metastasis surgery in Finland from 1997 to 2020 stratified by type of surgery (fusion vs non-fusion).

There were notable fluctuations in the annual incidence rates by primary cancer diagnosis. In 2017, the most common cancers were prostate (0.30 per 100,000 person-years) and breast (0.20 per 100.000 person-years) cancers (Figure 4). Both non-fusion and fusion surgery increased, with the 5-year rolling mean incidence of non-fusion surgery rising by 46%, while the incidence of fusion surgery increased by 53% (from 0.40 to 0.61) between 1999 and 2017 (Figure 5).

### Survival analysis

For the whole study period, the survival probabilities were 57% (CI 54–59) at 1 year, 44% (CI 42–46) at 2 years, 28% (CI 26–30) at 5 years, and 18% (CI 16–20) at 10 years (Table 2). The 1-year survival probability varied by age group, from 81% (CI 74–89) for patients under 40 years to 50% (CI 46–55) in patients aged 70–79 years (Table 3). Corresponding rates were 69% (CI 60–78) and 40% (CI 35–44) at 2 years.

**Table 2:** Kaplan–Meier survival analysis of all patients who underwent spinal metastasis surgery in Finland from 1997 to 2020 (n = 1,887) with a median follow-up of 1.30 (IQR 0.34–4.04) years

Follow-up	Number at risk	Survival probability % (CI)
1 year	1,013	57 (54–59)
2 years	744	44 (42–46)
5 years	393	28 (26–30)
10 years	167	18 (16–20)

IQR = interquartile range; CI = 95% confidence interval.

**Table 3:** Kaplan–Meier survival analysis of all patients who underwent spinal metastasis surgery in Finland between 1997 and 2020, categorized by 10-year age groups

Age	n	1-year follow-up		2-year follow-up		Follow-up, years median (IQR)
		Number at risk	Survival probability % (CI)	Number at risk	Survival probability % (CI)	
≥ 80	156	86	58 (51–66)	56	42 (35–51)	1.3 (0.4–3.0)
70–79	490	233	50 (46–55)	167	40 (35–44)	0.9 (0.2–3.1)
60–69	580	317	56 (52–60)	227	42 (38–46)	1.2 (0.4–4.0)
50–59	363	203	57 (52–63)	151	44 (39–49)	1.4 (0.4–3.9)
40–49	153	91	61 (53–69)	74	51 (44–60)	2.0 (0.5–6.7)
< 40	103	83	81 (74–89)	69	69 (60–78)	5.6 (1.4–13.7)

CI = 95% confidence interval; IQR = interquartile range.

The 1-year survival probability was highest in patients with primary breast cancer at 75% (CI 69–81) and lowest in patients with kidney cancer at 45% (CI 38–53) and prostate cancer at 47% (CI 42–53) (Table 4).

**Table 4:** Kaplan–Meier survival analysis of all patients who underwent spinal metastasis surgery in Finland in 1997 to 2020, categorized by primary cancers

Primary diagnosis	n	1-year follow-up		2-year follow-up		Follow-up, years median (IQR)
		Number at risk	Survival probability % (CI)	Number at risk	Survival probability % (CI)	
Breast	219	156	75 (69–81)	116	59 (52–66)	2.2 (0.8–4.8)
Kidney	169	72	45 (38–53)	39	25 (19–32)	0.8 (0.3–1.9)
Lung	58	33	57 (45–71)	22	45 (33–59)	1.3 (0.3–3.6)
Lymphoma	102	70	69 (61–79)	61	63 (54–73)	4.3 (0.6–9.5)
Myeloma	196	135	71 (65–78)	108	59 (53–67)	3.0 (0.6–6.6)
Other	817	415	53 (49–56)	311	42 (38–45)	1.1 (0.3–3.7)
Prostate	284	132	47 (42–53)	87	33 (28–39)	0.9 (0.3–2.7)

CI = 95% confidence interval; IQR = interquartile range.

## Discussion

We aimed to report incidence rates and survival after surgery for MSD based on nationwide register data. We found a significant increase in surgery for MSD between 1997 and 2020, while the estimated survival after surgery remained stable.

### Incidence rates

Previous studies have reported increased rates of surgery for metastatic spine disease [7,14]. Population-based data on the incidence of surgery is, however, limited. Wright et al. reported data from 22 specialized cancer centers on 3 continents [7]. The steepest increase in their surgery rates occurred earlier and was much greater than in the Finnish population, reflecting the pioneering role of these centers. In Finnish nationwide data, the upward turn in surgery rates for metastatic spine disease after the landmark study of Patchell et al. [4] was less pronounced. This finding raises the possibility of undertreatment, which could stem from multiple reasons: conservative attitudes among practitioners, distribution of patients across the country with inadequate spine expertise, and delays in treatment pathways that may prevent access to timely surgery.

The most pronounced increase in surgeries occurred in patients aged 70–80 years, owing to the increasing cancer burden in this age group. Recent studies also advocate surgery for the elderly [6]. Developments in less invasive instrumentation and access techniques have made surgery tolerable for more frail patients [5]. However, according to the authors, minimally invasive stabilization techniques did not surface in Finland during the study period. Although surgical rates remained low among the oldest age groups in Finland, the proportional increase in this cohort was clear. The most common primary pathologies, breast and prostate cancer, aligned with trends observed in other Western populations [7,9].

Compared with some other Western regions, Finland has been conservative in adopting new technologies and ideas in spine surgical practice. While the focus elsewhere has shifted toward preventing metastatic spinal cord compression, such as through proactive stabilization of lytic lesions, these practices appear less prevalent in Finland as indicated by the surprisingly low proportion of fusion surgeries (40–60% in recent years). Instrumentation rates of up to 70% have been reported in Europe [8,15]. Our findings suggest that patients with MSD have ended up in hospitals with limited capabilities for immediate instrumentation. In addition, especially without less invasive instrumentation options, patients may have been deemed fit only for palliative laminectomy. Furthermore, advancements in radiotherapy regimens during the study period may have improved local progression-free survival and altered the need for surgery [16,17].

### Survival analysis

Short-term survival rates throughout the study period (mean 6-month survival between 57% and 76%) were comparable to those reported in Sweden (median estimated survival of 6.2 months), where also the most common indication for surgery was neurologic deficit [8]. A patient series from a German center that included more stabilization surgeries and vertebral augmentation procedures demonstrated a higher survival rate (median survival of 18.4 months) [15]. Previous data indicates that spinal cord compression, neurologic deficits, and poor performance status are linked to shorter survival [18]. In this study, younger age and lymphoma as the primary cancer projected the best long-term survival, which was higher than anticipated (28% at 5 years and 18% at 10 years). This may be secondary to patient selection (e.g., performing surgery in lymphoma patients when radiation therapy could have sufficed and applying too strict a threshold for surgery in more frail patients). Advancements in medical oncology continue to improve survival in metastatic cancer and may alter the order of prognosis among primary diseases. Although the prognosis for kidney cancer has generally improved recently, postoperative survival in the MSD cohort remained poor in our data [19].

### Limitations

Register data lack detailed information on surgeries, the clinical presentations prompting surgeries, and the reason for exclusion from surgical intervention. Therefore, we cannot estimate how well the performed surgeries have met the actual needs of the population. Overall, there are no universal criteria for spine surgery in MSD populations. This prevents inferring causes for the observed trends. The Finnish Cause of Death register covers all deaths of people living in Finland, with only death data after emigration missing, thus potentially overestimating survival, which we consider an extremely marginal limitation in the metastatic cancer population. Although the generally high coverage and accuracy of the registers used [11-13] are strengths of this study, it remains possible that miscoding in individual cases may have introduced minor bias. The low number of surgeries for disc herniation or spondylosis excluded after formation of the study sample (42 of 1,887 patients) supports that our sample is representative of MSD surgeries. Although not directly generalizable to other countries, the present nationwide data provides unique perspectives on spinal metastasis surgery, highlighting potential treatment gaps that may not be visible in reports from specialized centers. Our data emphasizes the continuous need to ensure equal and adequate access to high-quality care.

### Conclusion

Surgery for metastatic spine disease in Finland increased moderately between 1997 and 2020. During this period, overall survival rates remained relatively stable. MSD surgery was primarily an adjunct treatment in the end-of-life phase for cancer patients in Finland.

### Supplementary data

Tables S1 and S2 are available as Supplementary data on the article page, doi: 10.2340/17453674.2025.43264

## Supplementary Material



## Notes

LT planned the study and drafted the manuscript. VP performed the statistical analyses. All authors revised the manuscript and accepted its final version.

Handling co-editors: Paul Gerdhem and Philippe Wagner

*Acta* thanks Christian Carrwik and other anonymous reviewers for help with peer review of this manuscript.
