# Supplementary material for: Incidence of and survival after surgery for metastatic spine disease: a nationwide register-based study between 1997 and 2020 from Finland
Source: Acta Orthop. 2025 Mar 10;96:250–5. doi: 10.2340/17453674.2025.43264 (PMC11894730; doi:10.2340/17453674.2025.43264)
Supplement: Supplementary file 1 [file ActaO-96-43264-s1.pdf]

Supplementary data

Table S1. The NOMESCO procedural codes used to collect the spine surgeries

Procedural codes	Procedure(s)
**Fusion surgeries**	
ABC21, NAG40, NAG41, NAG42, NAG50, NAG51, NAG52, NAG53, NAG56, NAG57, NAG61, NAG62, NAG63, NAG65, NAG66, NAG67, NAG99	Fusion surgeries
NAJ00, NAJ10, NAJ12, NAJ20, NAJ22, NAJ30, NAJ32, NAJ99	Fracture surgeries of the spine
**Non-fusion surgeries**	
ABB00, ABB02, ABB04, ABB06, ABB10, ABB20, ABB30, ABB40, ABB90, NAR00, NAR10, NAR20, NAR400, NAR99, NAR00, NAR10, NAR20, NAR40, NAR99	Tumor resections
ABC01, ABC04, ABC07, ABC10, ABC13, ABC16, ABC17, ABC20, ABC23, ABC26, ABC30, ABC33, ABC36, ABC40, ABC50, ABC53, ABC56, ABC60, ABC63, ABC66, ABC99	Decompression surgeries
NAK10, NAK20, NAK40, NAK99	Bone surgeries of the spine
NAS00, NAS20, NAS99	Infection surgeries of the spine
ABA00, ABA20, ABA30, ABA99, NAA32, NAB90, NAB92, NAC92, NAG70, NAG72, NAH10, NAH20, NAH30, NAH35,	Other spine surgeries

NAH60, NAT00, NAT10, NAT12, NAT14,

NAT20, NAT50, NAT99, NAU10, NAU20,

NAU99, NAW00, NAW10, NAW99

Table S2. Procedural and diagnosis codes at the index surgery used as exclusion criteria for the study

**Procedural codes used as exclusion criteria**	
ABB02, ABB04, ABB06, ABB20, ABB40,	Non-relevant tumor procedures
ABC01, ABC04, ABC07, ABC10, ABC13, ABC16, ABC17, ABC20, ABC23, ABC26,	Disc surgeries
NAS00, NAS20, NAS99	Infection surgeries
ABA20, ABA30, ABA99, NAB90, NAB92, NAC92, NAH10, NAH20, NAH30, NAH35, NAH60, NAT00, NAT10, NAT12, NAT14, NAT20, NAT50, NAT99, NAU10, NAU20, NAU99, NAW00, NAW10, NAW99	Other surgeries
**Diagnosis codes used as exclusion criteria**	
M47.2, M50.0, M50.1, M51.1, G55.1, G55.2	Disc herniation or spondylosis
C41.2, C41.8, C41.9	Primary cancer of the vertebral column
C70.1, C70.9	Primary cancer of spinal meninges
C72.0, C72.1, C72.8, C72.9	Primary cancer of the spinal cord or cauda equina
